# gRASping Depolarization: Contribution of RAS GTPases to Mitotic Polarity Clusters Resolution

**DOI:** 10.3389/fcell.2020.589993

**Published:** 2020-10-15

**Authors:** Roberto Quadri, Sarah Sertic, Marco Muzi-Falconi

**Affiliations:** Dipartimento di Bioscienze, Università degli Studi di Milano, Milan, Italy

**Keywords:** depolarization, Ras, mitosis, GTPase, phosphorylation, *Saccharomyces cerevisiae*

## Introduction

During their growth, all living cells undergo a process of polarization, defined as an asymmetric deposition and confinement of molecules and cellular functions. Much effort has been put into understanding how polarization is achieved and maintained and how it can be artificially induced, a field that has also been fueled by the fact that loss of polarity is a prerequisite for tumor development (Royer and Lu, 2011). This led to a vast comprehension of the mechanisms underlying polarity establishment and of the molecular components involved. On the other hand, we have limited knowledge on how polarity clusters are resolved when they are no longer necessary and what happens when this process fails. Here, we integrate our findings on polarity dispersion in budding yeast with literature evidence for a mitotic role of Ras proteins. We then propose a unifying view of how this GTPase might drive depolarization by direct recruitment of polarity factors.

## Dealing With Cellular Polarization

Polarization is a key event in cell life, as it allows the cell to compartmentalize the different features that are required for its growth, differentiation, and for the development of the whole organism. In all eukaryotes, polarity is controlled by the essential small GTPase Cdc42 (Etienne-Manneville, 2004), and cells direct polarized growth by spatial modulation of Cdc42 activity. A versatile tool to regulate the distribution of Cdc42-GTP in budding yeast is represented by the relocalization of its main GEF Cdc24 (Zheng et al., 1994; Caviston et al., 2002). In late G1, Cdc24 is found at the presumptive bud site, thus contributing to bud emergence; whereas in S and M phases, it accumulates at polarity clusters accounting for the growth of the daughter cell, before being sequestered in the nucleus in late mitosis (Nern and Arkowitz, 1999, 2000). Until now, most of the scientific efforts have focused on the way Cdc24 accumulation at the presumptive bud site drives bud emergence and growth. However, besides the relevance of polarity establishment, the polarization machinery must eventually be dispersed throughout mitosis to allow relocation of cellular factors and functions. We have reported a role for Haspin kinase in promoting depolarization in budding yeast. Exploiting haspin mutants, we have identified the consequences of failures in such process, which dooms the cells to death upon mitotic delays (Panigada et al., 2013). We recently built up on these data to identify the underlying pathway, unveiling the pivotal contribution played by Ras to the dispersion of polarity clusters (Quadri et al., 2020).

## Shaping the Cell Through GTP-Ras: Evidence From Cdc24

Ras is a eukaryotic small GTPase with a prominent role in cell-cycle commitment. In particular, it integrates intracellular and extracellular signals (e.g., nutrient availability or growth factors) to trigger cellular proliferation (Stacey and Kazlauskas, 2002). Accordingly, hyperactivation of Ras pathway is frequently observed in tumors (Fernández-Medarde and Santos, 2011), where it drives the growth of the malignant mass and resistance to apoptosis (Cox and Der, 2003). As a consequence, this GTPase has long been studied with regard to its high relevance in cell proliferation and carcinogenesis (Murugan et al., 2019).

We have recently reported a novel contribution of Ras to mitotic depolarization in budding yeast cells (Quadri et al., 2020), where it acts as a part of a bipartite pathway differentially regulating localization of Cdc24 during the cell cycle. In early stages of the cell cycle, Cdc24 binds to Bem1 and Rsr1 at the presumptive bud site (Butty et al., 2002; Park et al., 2002), where it promotes clustered Cdc42 activity leading to bud emergence and growth (Woods et al., 2015). Highlighting the exclusive role played by Bem1 and Rsr1 in the budding process, *bem1*Δ*rsr1*Δ mutants are virtually unviable (Irazoqui et al., 2003) (with few exceptions that possibly reflect a minor contribution by other proteins; Smith et al., 2013; Woods et al., 2015). Later in mitosis, polarized Cdc24 has to be dispersed (Gulli et al., 2000; Quadri et al., 2020), causing the redistribution of Cdc42-GTP to the whole daughter PM. Failure of this process leads to a persistence of polarity clusters (Quadri et al., 2020), potential nuclear missegregation, and cell death (Panigada et al., 2013). Thus, a system that couples the formation and resolution of polarity clusters to cell-cycle progression must be present. A convenient mechanism would be a switch in binding partners of Cdc24 upon reversible cell-cycle–dependent posttranslational modifications. The idea of a dependence of Cdc24 localization on its PTMs was first proposed by Gulli et al. (2000). The article reports a strong preferential binding of Bem1 to hypophosphorylated Cdc24 and a Bem1-dependent bud tip hyperaccumulation of the GEF upon failures in its phosphorylation (Gulli et al., 2000). Consistently, Cdc24 phosphorylation peaks after bud emergence, and Cdc28-Cln and the PAK Cla4 were identified as the kinases responsible for such PTMs (Gulli et al., 2000; Bose et al., 2001; Wai et al., 2009; Rapali et al., 2017). However, until now, the change in localization of the GEF was seen as a mere dissociation from the bud tip in late stages of the cell cycle, and no roles for this process were described. We have recently shown that mitotic Cdc24 is actively redistributed from the bud tip to the whole daughter PM in a phosphorylation-dependent manner. Bem1 and Rsr1 are completely dispensable to this process, which rather relies on a direct physical interaction of Cdc24 with GTP-loaded Ras (Quadri et al., 2020), which is evenly distributed by vesicles to the PM in mitosis (Quadri et al., 2020). By relocalizing Cdc24, this pathway redistributes Cdc42 activity from the bud tip to the whole PM, ultimately promoting depolarization. When this mechanism is impaired, cells accumulate polarity factors at the bud tip that, in case of mitotic delays, leads to unbalanced nuclear segregation and cell death (Quadri et al., 2020). Following polarity clusters removal, at the time of cytokinesis, Cdc24 is dephosphorylated (Bose et al., 2001), likely disrupting Ras interaction and making it available for the next cell cycle.

Our findings integrate Gulli's hypothesis that mitotic phosphorylation of Cdc24 by Cla4 acts as a molecular switch to modulate its physical interactions. Accordingly, mitotic cells lacking Ras are characterized by a diffused, cytoplasmic Cdc24 with only a residual accumulation of the GEF at the bud tip (Quadri et al., 2020). This excludes a competition between Ras and Bem1/Rsr1 in favor of a change in the GEF interactors upon its phosphorylation. Noteworthy, Cdc24, Cdc42-GTP, Bem1, and Cla4 have been reported to constitute a positive feedback loop to build robust polarity clusters promoting symmetry breaking and bud emergence in G1 (Howell and Lew, 2012; Witte et al., 2017). However, our results (Quadri et al., 2020), along with previous findings (Gulli et al., 2000; Rapali et al., 2017), support a bipartite role of this complex, with a second, negative feedback loop promoting polarisome dispersal later in the cell cycle. The molecular switch that triggers Cla4 activity toward Cdc24 is still to be elucidated, but likely resides in a priming phosphorylation event on the GEF by a G2/M-specific kinase. An ideal candidate might be Clb-coupled Cdc28, as it promotes the switch from apical to isotropic growth (Lew and Reed, 1993), and mutants that fail to activate Cdc28-Clb kinase accumulate Bem1-bound Cdc24 at the bud tip (Gulli et al., 2000).

## Extending the Model: A Mitotic signature for Ras-GTP Binding and Cellular Depolarization in Yeast

A similar system has been described to regulate the localization of Lte1, a putative GEF that takes a non-essential part in the mitotic exit network (Falk et al., 2011) and shows an impact on polarity in budding yeast (Geymonat et al., 2009, 2010). The pattern and mechanism regulating Lte1 distribution along the cell cycle exhibit remarkable analogies with those of Cdc24, possibly highlighting a common mean to drive mitotic relocalization of polarized proteins to the PM. Recruitment of Lte1 to the bud tip in early cell-cycle stages depends on a physical interaction with a polarisome component, Kel1 (Seshan et al., 2002; Gould et al., 2014). Similarly to Cdc24, Lte1 is phosphorylated by Cla4 and Clb-Cdc28 (Seshan and Amon, 2005; Geymonat et al., 2010), and overexpression of *CLA4* is sufficient to promote recruitment of Lte1 to the bud cortex in the absence of Kel1 (Seshan et al., 2002). This suggests that the phosphorylation of Lte1 acts as a molecular switch to promote binding to different cortex scaffolds. Accordingly, later works have shown that phosphorylation by Cdc28 and Cla4 primes Lte1 for direct physical interaction with GTP-Ras (Yoshida et al., 2003; Seshan and Amon, 2005; Geymonat et al., 2009, 2010), leading to its accumulation along the bud cortex. Similarly to Cdc24, at the end of mitosis dephosphorylation of Lte1 leads to its dispersion in the cytoplasm (Jensen et al., 2002; Seshan et al., 2002). Although the exact contribution of Lte1 to depolarization has not been unveiled, the observation that Lte1 mutants defective for Ras-binding experience hyperpolarized growth (Geymonat et al., 2010) highlights the role of the GTPase in promoting polarity cluster dissolution.

**Figure 1:**
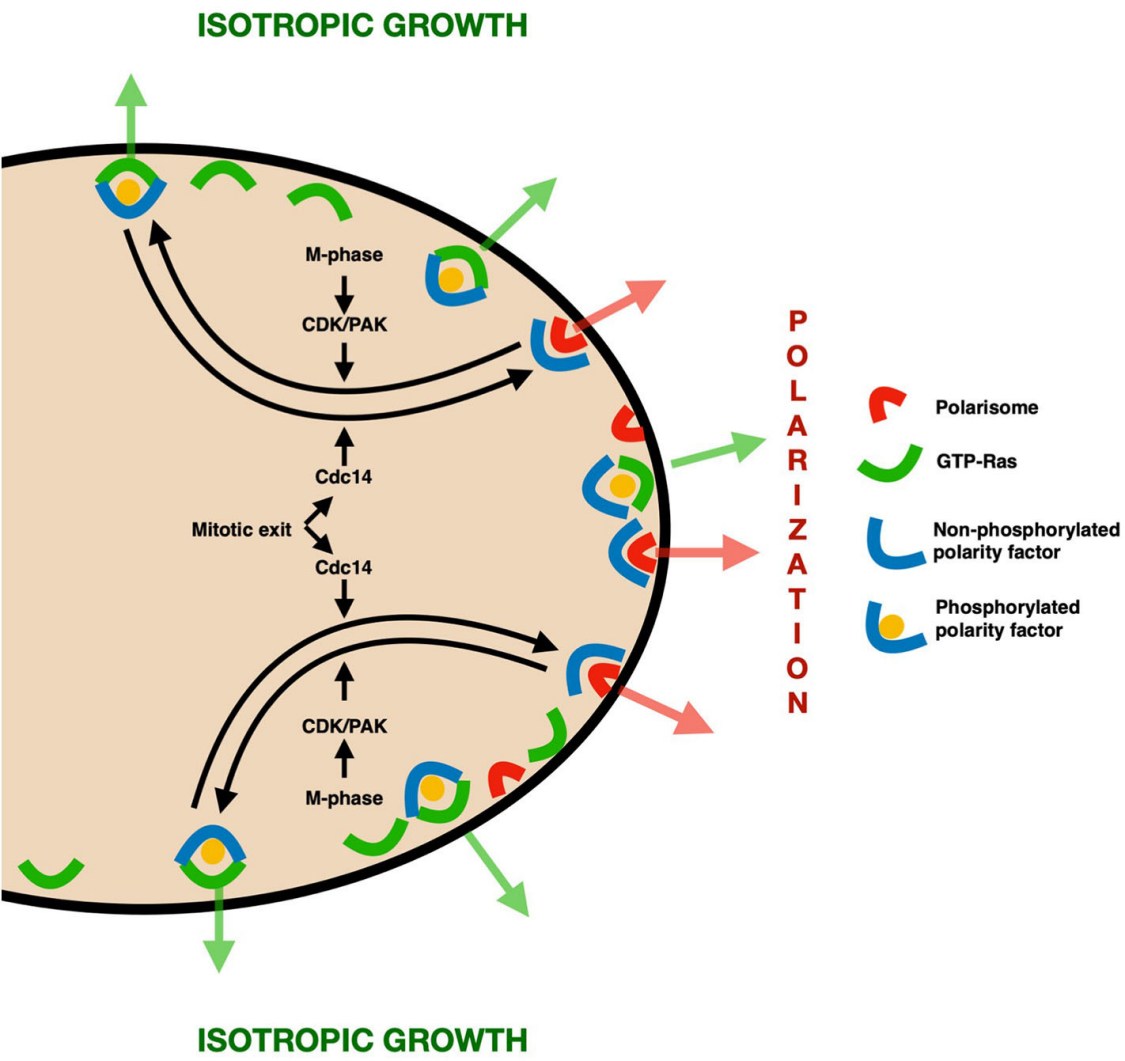
Model—In the early stages of the cell cycle, bud emergence and growth are promoted through the accumulation of polarity factors (e.g., Cdc24 and Lte1) at the presumptive bud site and later on at the bud tip, thanks to physical interaction with polarisome components. At the time of G2/M phase, however, some of these proteins need to be redistributed to the whole daughter cortex to prevent the detrimental effects of hyperpolarization. This redistribution is regulated by Cdc-Clb/PAK–mediated phosphorylation of such polarity factors, which acts as a molecular switch to promote their binding to the evenly distributed GTP-Ras, thereby leading to isotropic growth. At the end of the cell cycle, the phosphatase Cdc14 removes the phosphate groups, replenishing the cellular pool of polarization-promoting proteins.

A common scheme thus emerges from these observations (Figure 1). Proteins (possibly bearing a GEF-like domain) that take part in polarization first accumulate at the presumptive bud site by physical interaction with components of the polarisome. By the time of mitosis, however, the polarity clusters have to be redistributed to promote an isotropic growth and prevent detrimental hyperpolarization. To this end, a convenient docking site is provided by GTP-loaded Ras, which is at this stage evenly distributed to the whole PM (Quadri et al., 2020). We propose that the molecular switch that regulates this change in interactions is represented by phosphorylation events performed by Clb-Cdc28 and the kinase Cla4, whose activity is coupled to late stages of the cell cycle, thus preventing unscheduled depolarization. At the end of the cell cycle, mitotic phosphorylation is removed by Cdc14, detaching polarisome components from GTP-Ras and making them available for a new cell cycle.

## Ras Contribution to Cell Shape in Other Organisms

All the components of the pathway described in *Saccharomyces cerevisiae*, namely, Ras, Cdc42, and its GEFs and PAK, are conserved throughout the eukaryotic lineage. Several lines of evidence suggest that similar mechanisms might promote mitotic depolarization in other eukaryotes.

Links between Ras and polarity have been reported in *Schizosaccharomyces pombe* and *Cryptococcus neoformans* (Chang et al., 1994; Nichols et al., 2007), where a physical interaction between GTP-Ras and Cdc24 has been observed. However, although hyperpolarization has been observed in Ras mutants (Ballou et al., 2013), the GTPase seems to be mainly related to polarity establishment rather than to the resolution of polarity clusters.

A major difference between interphase and most mitotic animal cells is represented by the loss of cellular protrusions, substrate attachments, and cell–cell interactions observed during mitotic roundup (Théry and Bornens, 2008). In this scenario, Cdc42 is required to regulate the actin cytoskeleton and determine mitotic spindle orientation, which in turn will define the polarity axis of the daughter cells (Jaffe et al., 2008). Although the underlying mechanisms are still to be elucidated, alterations in Ras pathway impact on spindle orientation (Tang et al., 2011). Moreover, multiple high-throughput screenings identified physical interactions between RAS and CDC42 regulators and effectors (Adhikari and Counter, 2018; Steklov et al., 2018; Kovalski et al., 2019), including several RHO GEFs with putative activity for CDC42, although none of these have been validated yet.

On the other hand, the idea that mitotic redistribution of Cdc42 activity drives cellular depolarization is backed by multiple observations. Indeed, several studies highlight a loss of cellular polarity upon increased Cdc42 activity in multiple systems (Florian et al., 2012; Gao et al., 2019). Although it is not clear whether the observed phenotype is induced by an active depolarization mechanism or a deficient polarization machinery, this clearly demonstrates that a diffuse Cdc42 activity might be a mean to counteract cellular polarization. Moreover, a previous work in *Drosophila melanogaster* reported a redistribution Cdc42 to achieve a homogenous PM localization in mitosis (Rosa et al., 2015). The authors also reported that overexpression of the Pbl Cdc42 GEF leads to a diffuse relocalization of a Cdc42-containing polarity complex in non-mitotic cells, suggestive of a GEF-based mechanism to induce cellular depolarization in this stage of the cell cycle.

Although such evidence does not directly infer the existence of a mechanism for depolarization based on Ras-dependent mitotic redistribution of Cdc42 activity, it suggests that a similar network might be present also in other eukaryotes.

Mitotic cellular depolarization results from the integration of multiple pathways that ensure proper cell division and that share some remarkable features with the proposed Ras-based mechanism. The planar cell polarity (PCP) is a network active in epithelial cells that detects environmental cues and transduces them in a tissue-homogeneous planar polarization (Butler and Wallingford, 2017). The establishment of this polarity axis is granted in interphase by a differential accumulation of PCP components at opposite domains with distinct functions. However, during mitosis, PCP clusters must be resolved to avoid disruption of tissue polarity (Devenport et al., 2011). This process is promoted by the mitotic kinase Plk1, which phosphorylates the PCP subunit Celsr1 (Shrestha et al., 2015), priming it for internalization by endocytosis (Devenport et al., 2011; Heck and Devenport, 2017). Thus, it appears that phosphorylation of polarity factors by mitotic kinases and vesicle-driven mechanisms might be a conserved way to couple cell-cycle progression with resolution of polarity clusters. Additional studies will be required to elucidate this possibility and to dissect the contribution of Ras to mitotic depolarization in higher eukaryotes, eventually lighting a path for further Ras targeting to tackle cancer progression.

## Author Contributions

RQ and MM-F interpreted the data and wrote the paper. SS contributed to data interpretation and discussions. All authors contributed to the article and approved the submitted version.

## Conflict of Interest

The authors declare that the research was conducted in the absence of any commercial or financial relationships that could be construed as a potential conflict of interest.
